# Effects of a Web-Based Educational Program Regarding Physical Restraint Reduction in Long-Term Care Settings on Nursing Students: A Cluster Randomized Controlled Trial

**DOI:** 10.3390/ijerph18136698

**Published:** 2021-06-22

**Authors:** Eun-Hi Kong, Myoungsuk Kim, Seonho Kim

**Affiliations:** 1 College of Nursing, Gachon University, Seongnamdaero, Sujeong-gu, Seongnam 13120, Korea; ehkong@gachon.ac.kr; 2 College of Nursing, Kangwon National University, Chuncheon 24341, Korea; 3 Department of Nursing Science, College of Medicine, Chungbuk National University, Chungdae-ro 1, Seowon-gu, Cheongju 28644, Korea; sunhkim@chungbuk.ac.kr

**Keywords:** students, nursing, education, distance, restraint, long-term physical care, randomized controlled trial

## Abstract

Physical restraint is still frequently used in many countries. However, a lack of education hinders physical restraint reduction in long-term care facilities. No study has yet to examine the effects of physical restraint reduction education on nursing students. This study aimed to evaluate the effects of a web-based educational program of physical restraint reduction on nursing students’ knowledge and perceptions. A cluster randomized controlled and single-blind design was used. This study was conducted at four nursing schools in South Korea. A total of 169 undergraduate nursing students completed this study. Using random allocation, two nursing schools (85 students) were allocated as the experimental group and the other two schools (84 students) as the control group. The experimental group received the web-based educational program, and the control group did not receive the educational program. Data were collected immediately before and after the web-based educational program. The experimental group’s knowledge and perceptions significantly improved between pre-test and post-test. The analysis of covariance showed statistically significant differences between groups in knowledge (*p* < 0.001) and perceptions (*p* < 0.001) over time, revealing positive effects of the web-based educational program. The web-based educational program regarding physical restraint reduction positively affected nursing students’ knowledge and perceptions. Future studies are required to examine the educational program’s longitudinal effects with more rigorous measurements and research methods.

## 1. Introduction

Physical restraint is defined as “any action or procedure that prevents a person’s free body movement to a position of choice and/or normal access to his/her body by the use of any method, attached or adjacent to a person’s body that he/she cannot control or remove easily” (p. 2309) [1]. Despite many adverse effects and fatalities [2], the prevalence of physical restraint use in long-term care facilities is still high in many countries: 84.9% in Spain [3], 78.2% in South Korea [4], 70.2% in Hong Kong [5], 26.8% in Switzerland [6], 25.8% in China [7], and 19.6% in Germany [8].

Most Asian countries face rapidly increasing numbers of people with dementia, low-quality care in long-term care facilities, and a high prevalence of physical restraint use [4,5,7,9]. In Korea, physical restraints are commonly used in most long-term care facilities. Furthermore, many Korean long-term care facilities still frequently use many kinds of physical restraints (i.e., bedside rails, mitts, wrist restraints, trunk restraints, or chairs to prevent rising) [4].

Most existing studies regarding physical restraint reduction targeted staff in long-term care facilities [10,11,12,13,14]. According to an earlier study regarding the barriers to physical restraint reduction [10], many staff had an unclear definition of restraint and insufficient education about physical restraint reduction. Staff tended to use restraints in unclear situations and based on their experiences rather than from evidence-based guidelines [11,12,13]. Earlier review studies [10,11,14] reported urgent needs of education and the positive effects of educational training on restraint reduction in long-term care facilities.

Globally, there is a lack of physical restraint studies targeting nursing students [15,16,17]. Only a few studies have explored nursing students’ knowledge and attitudes related to physical restraint in acute care or psychiatric care settings using a survey method [15,16,17]. One study [15] explored Israeli nursing students’ attitudes toward physical restraint in acute care settings. Another study [16] compared nursing students’ and non-nursing students’ attitudes regarding containment measures for psychiatric patients in the United Kingdom. The other study [17] compared knowledge and attitude between nursing students and nurses regarding physical restraints for older adults in hospitals.

According to existing literature [18,19,20], nursing students showed insufficient knowledge, misconceptions, and inappropriate preparedness about the care of older adults in long-term care settings around the world. Despite the rapidly increasing elderly population and long-term care facilities, gerontological nursing education in many countries, especially in Asia, lacks content related to restraint-free care in long-term care facilities. Furthermore, there is a dearth of research that focused on nursing students regarding physical restraint in geriatric long-term care settings globally. Only one qualitative study [20] explored nursing students’ experiences related to physical restraint use in Korean long-term care facilities. According to the qualitative study [20], nursing students had an unclear definition of physical restraint, observed common uses of physical restraint, were asked to help nursing home staff in using physical restraint, and expressed the need for physical restraint reduction education.

Web-based education is commonly used in nursing education globally due to the rapid growth of internet usage. Earlier review studies [21,22] reported significant positive effects of web-based education on nursing students in terms of knowledge acquisition and clinical performance. According to previous studies, nursing students perceived the benefits of web-based learning [23] and showed high satisfaction with web-based education [22]. Korea is famous for its high-access and high-speed internet service around the world. According to the Organization for Economic Co-operation and Development (OECD)’s survey, 99.7% of all households in South Korea have internet access [24]. Therefore, Korean students have high readiness and acceptance for web-based learning.

No study has yet explored the effects of nursing students’ educational programs for physical restraint reduction. Therefore, this study aimed to explore the effects of a web-based educational program for physical restraint reduction on nursing students’ knowledge and perceptions. In addition, this study explored nursing students’ satisfaction with the web-based educational program for physical restraint reduction.

## 2. Methods

### 2.1. Aim

This study aimed to explore the effects of a web-based educational program regarding physical restraint reduction in long-term care facilities on the knowledge and perceptions of fourth-year nursing students in undergraduate nursing programs. In addition, students’ satisfaction with the web-based educational program was examined.

### 2.2. Study Design

The study was a prospective cluster randomized controlled trial with a pre-test–post-test experimental design. Four nursing schools were randomly assigned to either the experimental group or the control group using opaque allocation envelopes by a nursing researcher who was not involved in this study. Research assistants and participants were blind to the assignment. Data were collected from the two groups at two time points (before intervention and immediately following intervention) between November 2015 and January 2016. This study followed the Consolidated Standards of Reporting Trials (CONSORT) guideline [25].

### 2.3. Sample, Setting and Recruitment

Using a convenience sampling method, the primary author contacted six nursing schools located in four provinces in South Korea, which consists of nine provinces. Two schools declined to participate due to students’ participation in another study and their busy schedules. Finally, the participants were recruited from four nursing schools located in three provinces of South Korea.

The four nursing schools met the following inclusion criteria: (a) provided gerontological nursing course and gerontological nursing practicum course; (b) lacked an educational program of physical restraint reduction in long-term care facilities; and (c) had no plan to provide physical restraint reduction education within the next 4 months. Inclusion criteria for individual participants comprised the following: (a) junior- or senior-level students; (b) had taken gerontological nursing practicum; (c) had never received education about physical restraint reduction in long-term care facilities; (d) had the experience of observing physical restraint use in long-term care facilities; and (e) understood the purpose and process of this study and agreed to participate in this study. Exclusion criteria of the participants included the following: (a) had difficulties in using the internet; and (b) had the experience of participating in similar research.

**Figure 1:**
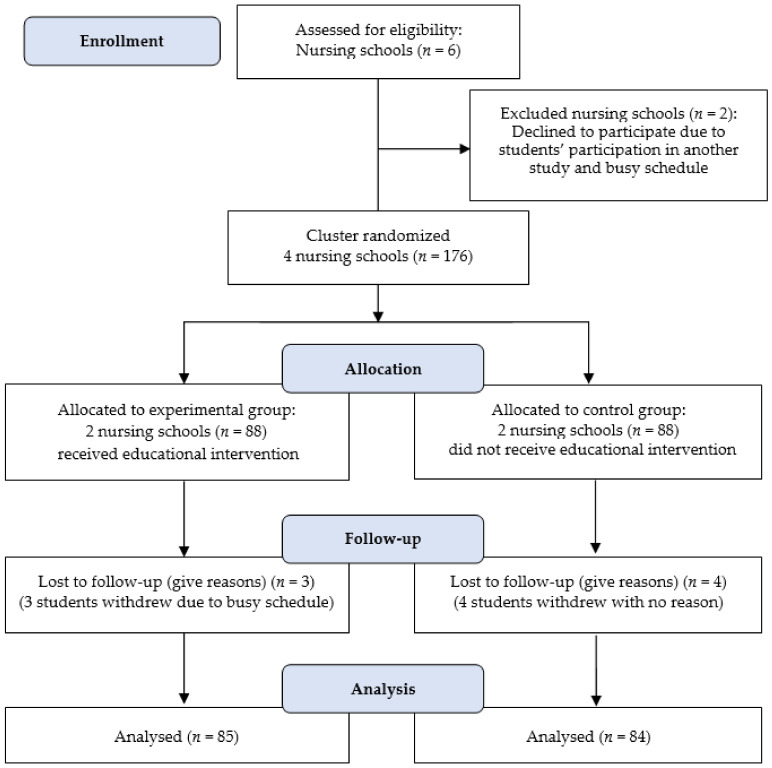
A flow diagram of participant recruitment and retention throughout the study.

The sample size was calculated (number of groups = 2, α = 0.05, power = 0.8, number of measurements = 2, and effect size = 0.11) using G* power 3.0.10 software (Franz Faul, Kiel, SH, Germany). The effect size was estimated based on a relevant earlier study [11]. The minimum number of participants was 166 (88 in each group), but 176 people were enrolled, considering a 6% dropout rate. A total of 176 nursing students participated in this study, and 7 of them dropped out due to their busy schedules or unknown reasons (Figure 1) [25]. Finally, 169 nursing students (85 in the experimental group and 84 in the control group) completed this study (Figure 1) [25].

### 2.4. Intervention

The intervention was one self-directed web-based educational program for physical restraint reduction in long-term care facilities, which was developed and evaluated with nursing home staff (nurses, nurse aides, and care workers) in previous studies [26,27]. The web-based educational program consisted of the following six video sessions (a total of 54.33 min): (1) definition, prevalence, reasons, myths, and needs regarding physical restraint (6.42 min); (2) untoward effects, clients’ rights, and responses regarding restraint use (6.36 min); (3) individualized care approach and practice guide of physical restraints (11.01 min); (4) fall prevention and intervention (12.36 min); (5) individualized care for behavioral symptoms of people with dementia (8.07 min); and (6) residents interfering with treatment, physical restraint use, and legal issues (10.11 min) [26,27]. Only the experimental group received the web-based educational program. After completing data collection and analysis, the control group was invited to receive the same web-based educational program.

### 2.5. Ethical Considerations

Ethical approval for the research was obtained from the institutional review board (IRB) of the principal investigator’s university (IRB No. 1044396-201511-HR-055-01). After receiving IRB approval, the authors contacted department chairs and professors in charge of gerontological nursing courses of potential nursing schools for this study. With the professors’ help, the authors contacted potential participants and provided information about this study’s aim, process, their voluntary participation, right to withdrawal, benefits and risks, anonymity, and confidentiality. After the submission of informed written consent, participants voluntarily participated in this study. All data were stored on the author’s password-protected computers for confidentiality.

### 2.6. Outcome Measures

A total of four questionnaires were employed. The demographic data survey included age, gender, religion, frequency of internet use, the experience of web-based education, frequency of smartphone application usage, and attitude about physical restraint use in long-term care facilities. In addition, two scales (i.e., measures of knowledge and perceptions about physical restraint use) were administered before the intervention (pre-test) and immediately after the intervention (post-test). Also, the satisfaction scale of the web-based program was used among the experimental group after the intervention.

The Knowledge about Physical Restraints scale [28] was used to assess participants’ knowledge regarding physical restraint. The scale contains 18 items with scores of 1 (true) or 0 (false; total score: 0–18). In this study, three scale items (the 7th, 12th, and 16th items) were deleted due to their inappropriateness in the Korean long-term care situation. A higher score indicates more knowledge regarding physical restraint. The Korean version of this scale was used, and the KR-20 coefficients were 0.74 [29] and 0.72 [26] in earlier studies [26,29]. In this study, the KR-20 coefficient was 0.62.

The Perceptions of Restraint Use Questionnaire (PRUQ) [30] was used to measure participants’ perceptions about physical restraint use. The PRUQ consists of 17 items scored on a 5-point Likert scale ranging from 1 (not at all important) to 5 (most important; total scores: 17–85). A higher score means participants ascribe greater importance to the reasons for restraint use. The Korean version of the PRUQ [31] was used, and Cronbach’s α coefficients were 0.93 [31] and 0.92 [26] in earlier studies [26,31]. The Cronbach’s α coefficient for the PRUQ in this study was 0.91.

The Satisfaction of Web-Based Education scale [32] was used to measure participants’ satisfaction toward the web-based educational program. The satisfaction scale contains 10 items scored on a 5-point Likert scale ranging from 1 (entirely unsatisfactory) to 5 (entirely satisfactory; total score: 10–50). A higher score indicates more satisfaction with the web-based educational program’s contents. Cronbach’s α coefficients were 0.93 in an earlier study [32] and 0.87 in this study.

### 2.7. Data Analysis

Data were analyzed using the Statistical Package for the Social Sciences (IBM SPSS version 23.0, SPSS Inc., Chicago, IL, USA). The data were described with frequency, percentage, mean, and standard deviation. Pearson’s Chi-square test, Fisher’s exact test, and independent *t*-test were used, respectively, for categorical and continuous variables to test for differences between the two groups. The differences of dependent variables between groups were analyzed using an analysis of covariance (ANCOVA), with post-test scores as the dependent variable, group as the independent variable, and gender and pre-test scores as covariates for adjusting baseline differences. Two-tailed statistical analyses were used, and *p*-values less than 0.05 were regarded as statistically significant. Satisfaction with the web-based educational program was described based on the mean score and percentage of individual items.

## 3. Results

### 3.1. General Characteristics and Homogeneity Test

A total of 169 participants completed this study. All participants were nursing students at 4-year universities who took the gerontological nursing practicum course. Table 1 shows the descriptive statistics of participants’ general characteristics. Most participants were 20~29 years of age and female (Table 1). Many participants used the internet and smartphone applications frequently (more than 5 times per day) and had previous experience with web-based education. Most participants (87.5%) reported that physical restraint use was very necessary or necessary (Table 1). There were no statistically significant differences between groups in demographic characteristics except for gender (Table 1). There was a significant difference in the knowledge of physical restraint between groups at pre-test (*t* = −3.26, * p* = 0.001; Table 2).

### 3.2. Educational Effects on Participants’ Knowledge and Perceptions

Table 3 showed the differences of within-group pre- and post-test scores and between-group post-test scores. The within-group results showed the mean knowledge score after the web-based educational program increased by 3.51 (SD = 2.30) in the experimental group while it increased by 0.35 (SD = 1.59) in the control group. The results of ANCOVA while controlling for pre-test score and gender showed the experimental group’s knowledge significantly improved (increased) compared to the control group (*p* < 0.001; Table 3). The web-based educational program had a large effect (partial η^2^ = 0.398) on the experimental group’s knowledge.

The mean perceptions score after the web-based educational program decreased by 19.05 (SD = 12.65) in the experimental group while it decreased by 1.00 (SD = 7.30) in the control group. The results of ANCOVA while controlling for gender revealed the experimental group’s perceptions score significantly improved (declined) compared to the control group (*p* < 0.001; Table 3). The web-based educational program had a large effect (partial η^2^ = 0.341) on the experimental group’s perceptions.

### 3.3. Satisfaction toward the Web-Based Restraint Reduction Educational Program

The mean satisfaction score of the web-based educational program in the experimental group was 4.36 (SD = 0.45), which meant participants in the experimental group were satisfied with the educational program (Table 4). The highest-scoring (mean = 4.64, SD = 0.53) item of satisfaction scale was “contents were easy to understand” and the lowest-scoring (mean = 3.55, SD = 1.02) item was “easier to concentrate than offline education” among the experimental group (Table 4).

## 4. Discussion

This study aimed to evaluate the effects of educational programs regarding physical restraint reduction on nursing students in undergraduate nursing programs. Few studies have targeted nursing students regarding physical restraint around the world. Only a few survey studies have explored nursing students’ knowledge and attitudes regarding physical restraint in acute care settings [15,17] or psychiatric units [16] using different measurement scales. No other study has investigated the effects of an educational program about physical restraint reduction on nursing students. Therefore, this study is very meaningful as it is the first study to explore the effects of web-based educational programs regarding physical restraint reduction in long-term care facilities on nursing students.

In this study, most students (87.5%) reported that physical restraint use in long-term care settings was very necessary or necessary at the pre-test survey. The educational program in this study, however, statistically significantly improved nursing students’ knowledge and perceptions regarding physical restraint reduction in long-term care settings. In addition, the educational program had large positive effects on students’ knowledge (partial η^2^ = 0.398) and perceptions (partial η^2^ = 0.341) regarding physical restraint reduction. Given that earlier systematic review studies [11,14] found inconsistent results and a lack of report of effect size in the existing literature, the results of this study are very meaningful and important.

It is difficult to compare the results of this study with those of earlier studies because no earlier studies evaluated the effect of an educational program regarding physical restraint reduction on nursing students around the world. However, this study supported the result of an earlier qualitative study [20] that reported nursing students’ need for physical restraint reduction education. In addition, the results of this study support previous studies [26,27,33] that reported significant improvements in nursing home staff’s knowledge, perceptions, or attitudes after receiving the educational program about physical restraint reduction.

This study showed the web-based educational program is an effective educational program for gerontological nursing and gerontological nursing practicum courses. In terms of an educational method, the results of this study support an earlier systematic review study [22] that reported the positive effects of web-based learning on knowledge acquisition among nursing students. Also, the students in this study showed very high satisfaction toward the web-based educational program. The results also supported a previous review study [22] that reported high satisfaction scores toward web-based education among nursing students.

As strengths, in order to minimize contamination and bias, this study employed cluster randomized trial, single blinding method, and sufficient sample size. In addition, this study had no loss of cluster at follow-up after randomization and a similar loss to follow-up between groups. This study provided detailed descriptions of research methods and results (i.e., differences scores within the group as well as between groups). This study is very meaningful given that many nursing students had an unclear definition of physical restraint, observed common uses of physical restraint, and inadequate preparedness about caring for older adults in long-term care settings [18,19,20]. The results of this study added new knowledge to the existing literature related to restraint reduction education and future nurses’ education.

This study, however, had some limitations. This study could not use individually randomized trials, double-blinding, and independent recruiter, which might lead to methodological biases. Moreover, this study did not examine the longitudinal effects of the web-based educational program. Therefore, caution needs to be taken in the generalization of the results to other populations and settings.

Future studies are required to use more robust research method (i.e., individually randomized controlled trial and double-blinding method) and to examine the longitudinal effect in order to reduce bias. Although this study used the globally widely used scale of knowledge about physical restraints, the scale was developed not for nursing students but for care staff. Additionally, three items of the knowledge scale were deleted in this study due to their inappropriateness in Korean long-term care situations, which may have affected lower reliability of the scale in this study than in earlier studies [26,29]. Future studies need to develop and evaluate more reliable measurements regarding restraint reduction for Korean nursing students. In this study, many students’ high usage of internet and smartphone applications and Korea’s high-access and high-speed internet service [24] might have affected the positive results for the web-based educational program. The web-based educational program might not be usable for nursing students in some countries with low internet usage.

## 5. Conclusions

Despite many harmful effects, physical restraints are commonly used in long-term care facilities around the world, so education regarding physical restraint reduction for future nurses is very important. The web-based educational program regarding physical restraint reduction in this study was very effective in improving nursing students’ knowledge and perceptions regarding physical restraint reduction. Nursing students showed high satisfaction toward the web-based educational program. Future studies are required to examine the educational program’s longitudinal effects with more rigorous measurements and research methods.

## Figures and Tables

**Table 1:** Participants’ general characteristics and homogeneity test between groups (*n* = 169).

Characteristics	Categories	Experimental Group (*n* = 85)	Control Group (*n* = 84)	Total (*n* = 169)	*χ* ^2^	*p*
		*n* (%)	*n* (%)	*n* (%)		
Age (year)	20~29	84 (98.8)	84 (100.0)	168 (99.4)	0.99 ^†^	1.000
	30~39	1 (1.2)	0 (0.0)	1 (0.6)		
Gender	Female	83 (97.6)	75 (89.3)	158 (93.5)	4.85	0.028
	Male	2 (2.4)	9 (10.7)	11 (6.5)		
Religion	Protestant	20 (23.5)	26 (31.0)	46 (27.2)	6.82 ^†^	0.072
	Buddhism	6 (7.1)	2 (2.4)	8 (4.7)		
	Catholic	7 (8.2)	15 (17.8)	22 (13.1)		
	None	52 (61.2)	41 (48.8)	93 (55.0)		
Frequency of internet usage	≥5 times a day	42 (49.4)	39 (46.4)	81 (47.9)	0.35	0.841
	3~4 times a day	24 (28.2)	23 (27.4)	47 (27.8)		
	≤2 times a day	19 (22.4)	22 (26.2)	41 (24.2)		
Experience of web-based education	Yes	77 (90.6)	67 (79.8)	144 (85.2)	3.93	0.050
	No	8 (9.4)	17 (20.2)	25 (14.8)		
Frequency of smartphone application usage	≥5 times a day	77 (90.6)	78 (92.9)	155 (91.7)	1.05 ^†^	1.000
	3~4 times a day	7 (8.2)	6 (7.1)	13 (7.7)		
	1~2 times a day	1 (1.2)	0 (0.0)	1 (0.6)		
Attitude about physical restraint use in long-term care facilities	Very necessary	4 (4.7)	4 (4.8)	8 (4.7)	2.80 ^†^	0.240
	Necessary	74 (87.1)	66 (78.5)	140 (82.8)		
	Not necessary	7 (8.2)	14 (16.7)	21 (12.5)		
	Never necessary	0 (0.0)	0 (0.0)	0 (0.0)		

Values are *n* (%); Tested by Chi-squared test; ^†^ Fisher’s exact test.

**Table 2:** Homogeneity test for main outcome variables between groups (*n* = 169).

Variables	Experimental Group (*n* = 85)	Control Group (*n* = 84)	*t*	*p*
	Mean ± SD	Mean ± SD		
Knowledge	10.24 ± 2.08	11.15 ± 1.49	−3.26	0.001
Perceptions	54.80 ± 9.53	52.33 ± 9.90	1.65	0.101

Values are mean ± standard deviation; Tested by independent *t*-test.

**Table 3:** Educational effects on main outcome variables (*n* = 169).

Variables Group (*n* = 169)		Pre-Test	Post-Test	Difference (Post-Pre)	Main Effects ^†^		
		Mean ± SD	Mean ± SD	Mean ± SD	F	*p*	Partial η^2^
Knowledge	Experimental group (*n* = 85)	10.24 ± 2.08	13.76 ± 1.39	3.51 ± 2.30	109.30	<0.001	0.398
	Control Group (*n* = 84)	11.15 ± 1.49	11.51 ± 1.56	0.35 ± 1.59			
Perceptions	Experimental group (*n* = 85)	54.80 ± 9.53	35.74 ± 11.61	−19.05 ± 12.65	86.01	<0.001	0.341
	Control Group (*n* = 84)	52.33 ± 9.90	51.33 ± 9.20	−1.00 ± 7.30			

Values are mean ± standard deviation; ^†^ ANCOVA with controlling pre-test and gender in knowledge and gender in perceptions; as a measure of effect size, partial η^2^ = 0.01, 0.06, and 0.14 represent small, medium, and large effect, respectively.

**Table 4:** Satisfaction toward web-based educational program among experimental group (*n* = 85).

Items	Entirely Unsatisfactory	Unsatisfied	Fair	Satisfied	Entirely Satisfactory	Mean ± SD
	*n* (%)	*n* (%)	*n* (%)	*n* (%)	*n* (%)	
It was easier to concentrate than offline education.	2 (2.4)	11 (12.9)	26 (30.6)	30 (35.3)	16 (18.8)	3.55 ± 1.02
The goals of education are clearly presented.	1 (1.2)	0 (0)	6 (7.1)	42 (49.4)	36 (42.4)	4.32 ± 0.71
The contents of education are accurately explained.	0 (0)	0 (0)	3 (3.5)	40 (47.1)	42 (49.4)	4.46 ± 0.57
It is composed so that the contents of education can achieve the educational goal.	0 (0)	0 (0)	3 (3.5)	42 (49.4)	40 (47.1)	4.44 ± 0.57
The contents of education include the latest information.	0 (0)	3 (3.5)	12 (14.1)	38 (44.7)	32 (37.6)	4.16 ± 0.80
The contents of education are reliable.	0 (0)	1 (1.2)	2 (2.4)	34 (40.0)	48 (56.5)	4.52 ± 0.61
The contents of the education were easy to understand.	0 (0)	0 (0)	2 (2.4)	27 (31.8)	56 (65.9)	4.64 ± 0.53
The contents of the education include necessary information.	0 (0)	0 (0)	1 (1.2)	32 (37.6)	52 (61.2)	4.60 ± 0.52
The education adequately describes professional terms.	0 (0)	0 (0)	2 (2.4)	41 (48.2)	42 (49.4)	4.47 ± 0.55
The contents of the education are generally satisfactory.	0 (0)	0 (0)	2 (2.4)	39 (45.9)	44 (51.8)	4.49 ± 0.55
Total						4.36 ± 0.45

Values are *n* (%) or mean ± standard deviation.

## Data Availability

The data presented in this study are available on request from the corresponding author. The data are not publicly available due to privacy restrictions.
